# Hypothalamic mTOR Signaling Mediates the Orexigenic Action of Ghrelin

**DOI:** 10.1371/journal.pone.0046923

**Published:** 2012-10-09

**Authors:** Luís Martins, Diana Fernández-Mallo, Marta G. Novelle, María J. Vázquez, Manuel Tena-Sempere, Rubén Nogueiras, Miguel López, Carlos Diéguez

**Affiliations:** 1 Department of Physiology, Research Center of Molecular Medicine and Chronic Diseases (CIMUS), University of Santiago de Compostela-Instituto de Investigación Sanitaria (IDIS), Santiago de Compostela, Spain; 2 CIBER Fisiopatología de la Obesidad y Nutrición (CIBERobn), Santiago de Compostela, Spain; 3 Department of Cell Biology, Physiology and Immunology, University of Córdoba, Cordoba, Spain; 4 Instituto Maimónides de Investigaciones Biomédicas (IMIBIC), Córdoba, Spain; University of Cordoba, Spain

## Abstract

Current evidence suggests that ghrelin, a stomach derived peptide, exerts its orexigenic action through specific modulation of Sirtuin1 (SIRT1)/p53 and AMP-activated protein kinase (AMPK) pathways, which ultimately increase the expression of agouti-related protein (AgRP) and neuropeptide Y (NPY) in the arcuate nucleus of the hypothalamus (ARC). However, there is a paucity of data about the possible action of ghrelin on alternative metabolic pathways at this level. Here, we demonstrate that ghrelin elicits a marked upregulation of the hypothalamic mammalian target of rapamycin (mTOR) signaling pathway. Of note, central inhibition of mTOR signaling with rapamycin decreased ghrelin’s orexigenic action and normalized the mRNA expression of AgRP and NPY, as well as their key downstream transcription factors, namely cAMP response-element binding protein (pCREB) and forkhead box O1 (FoxO1, total and phosphorylated). Taken together, these data indicate that, in addition to previous reported mechanisms, ghrelin also promotes feeding through modulation of hypothalamic mTOR pathway.

## Introduction

Ghrelin is a peptidergic hormone mainly secreted by the stomach, the effects of which are exerted through the growth hormone secretagogue receptor 1a (GHS-R1a) [1]. Central ghrelin administration directly activates agouti-related peptide/neuropeptide Y (AgRP/NPY) and inhibits POMC neurons in the arcuate nucleus of the hypothalamus (ARC) [2]–[10], which ultimately stimulates feeding and decreases energy expenditure [8], [11], [12].

Recently, the molecular pathways upstream of these ARC-derived neuropeptides have been identified. Ghrelin activates hypothalamic Sirtuin1 (Sirt1)/p53 and AMP-activated protein kinase (AMPK) signaling pathways, leading to inhibition of *de novo* lipogenesis and increased fatty acid oxidation, which causes changes in mitochondrial respiration and increased production of reactive oxygen species (ROS) [2], [4]–[10] finally leading to activation of NPY/AgRP neurons. The molecular mechanisms linking ghrelin with the induction of *Agrp* and *Npy* gene expression have also been recently identified. It was reported that the hypothalamic homeobox domain transcription factor BSX regulates ghrelin’s stimulatory effect on *Agrp* and *Npy* gene expression in rats, an effect that involves an interaction with two other transcription factors: the phosphorylated cAMP response-element binding protein (pCREB) and forkhead box O1 (FoxO1), respectively [7], [13], [14]. However, in spite of this evidence, it is unclear whether selective modulation of hypothalamic lipid metabolism is the unique main effector downstream of AMPK or if alternative pathways might be involved.

Mammalian target of rapamycin (mTOR) is an evolutionarily conserved serine-threonine kinase that acts as a cellular sensor of changes in energy balance, growth factors, nutrients and oxygen [15]–[19]. mTOR is a component of at least two multi-protein complexes: mTOR complex 1 (mTORC1) and mTOR complex 2 (mTORC2) [15], [16], [19]. mTORC1 phosphorylates and modulates the activity of the serine/threonine ribosomal protein S6 kinase 1 (S6K1). In turn S6K1 phosphorylates and activates S6, a ribosomal protein involved in translation [15], [16], [19]. Recent evidence has shown that hypothalamic mTOR signaling plays a major role in modulating energy balance by responding to nutrient availability and the hormonal milieu [20]–[28]. Thus, central administration of hormones (i.e. leptin, thyroid hormone, ciliary neurotrophic factor (CNTF), bone morphogenetic protein 7 (BMP7)) or metabolites (i.e. leucine, α-lipoic acid) regulate feeding through modulation of hypothalamic mTOR [20], [22], [23], [28], [29].

On the basis of this evidence, the aim of our study was to assess if ghrelin actions on feeding might be mediated by specific modulation of mTOR signaling in the hypothalamus. We provide novel evidence that ghrelin promotes feeding by modulating the hypothalamic mTOR pathway.

## Materials and Methods

### Animals

All experiments were carried out in accordance with the guidelines of the Spanish Committee for Experiments on Animals. All procedures performed were also approved by the University of Santiago de Compostela Institutional Bioethics Committee, the Xunta de Galicia (Local Government) and the Ministry of Economy and Competitiveness with ID PS09/01880. Outbred male Sprague-Dawley rats (University of Santiago de Compostela Animal House) weighing between 250–300 g were housed individually under standard conditions with an artificial 12∶12 hrs light/dark cycle under constant humidity. Animals were allowed free access to tap water and fed with standard laboratory chow during the experimental periods, as described below.

### Intracerebroventricular Treatments

For the ghrelin acute experiments, male rats received either an intracerebroventricular (ICV) administration [28], [30]–[35] of vehicle (5 µl of saline) or ghrelin (5 µg = 1.5 nmol) in a total volume of 5 µl (*Bachem,* Bubendorf, Switzerland) [4], [5], [7], [8], [10], [36]. For the experiments with rapamycin rats received an ICV injection of vehicle (5 µl of DMSO) or rapamycin (50 µg in a total volume of 5 µl; *Sigma*; St Louis, MO, USA) [28] 30 minutes prior to ghrelin administration. We used 8–10 rats per group and the experiments were repeated at least twice; animals were treated at 09∶00 AM (one hour after the light cycle had commenced), when they were satiated. Rats were killed by cervical dislocation. The hypothalamus and the ARC (for western blotting) were immediately homogenized on ice to preserve phosphorylated protein levels or the whole brain (for studies of *in situ* hybridization) were dissected, and stored at −80°C until further processing. The hypothalamus was defined by the posterior margin of the optic chiasm and the anterior margin of the mammillary bodies (to the depth of approximately 2 mm) [5], [7], [28], [32], [34], [35], [37]. Dissection of the ARC was performed by micropunches under the microscope, as previously shown [28].

### Stereotaxic Microinjection of Ghrelin

Rats were placed in a stereotaxic frame (*David Kopf Instruments;* Tujunga, CA, USA) under ketamine/xylazine anesthesia. The ARC was targeted bilaterally using a 25-gauge needle (*Hamilton;* Reno, NV, USA) [28]. The injections were directed to the following stereotaxic coordinates: −2.8 mm anterior-posterior (one injection was performed in each ARC), ±0.3 mm lateral to bregma and 10.2 mm deep. Ghrelin (*Bachem, Bubendorf, Switzerland*; 1 µg in 1 µL of saline during 2 hours) or vehicle (1 µL of saline) were given. Rats were killed by cervical dislocation. The ARC (for western blotting) was dissected [28], immediately homogenized on ice to preserve phosphorylated protein levels and stored at −80°C until further processing.

### Western Blotting

Hypothalamic and ARC protein lysates were subjected to SDS-PAGE, electrotransferred on a PVDF membrane and probed with the following antibodies: mTOR, pmTOR Ser2448, pS6K1 Thr389, S6, pS6 Ser235/236 (*Cell Signalling*; Danvers, MA, USA), CREB, FoxO1, pCREB-Ser^129^ and pFoxO1-Ser^256^ (*Santa Cruz*; Santa Cruz, CA, USA) and β-actin (*Abcam*; Cambridge, UK) as previously described [5], [7], [8], [10], [28], [32], [34], [35].

### In situ Hybridization

Coronal brain sections (16 µm) were probed with specific oligonucleotides for AgRP (5′-CGA CGC GGA GAA CGA GAC TCG CGG TTC TGT GGA TCT AGC ACC TCT GCC-3′), and NPY (5′-AGA TGA GAT GTG GGG GGA AAC TAG GAA AAG TCA GGA GAG CAA GTT TCA TT-3′) as previously published [4], [5], [7], [10], [28], [30], [32], [34], [35].

### Statistical Analysis

Data are expressed as mean ± SEM in relation (%) to vehicle-treated rats. Statistic significance was determined by t-Student when two groups were compared or ANOVA and *post-hoc* two-tailed Bonferroni test when more than two groups were compared. *P*<0.05 was considered significant.

## Results

### Central Administration of Ghrelin Increased Food Intake and Activated mTOR Signaling in a Time-dependent Fashion

**Figure 1:**
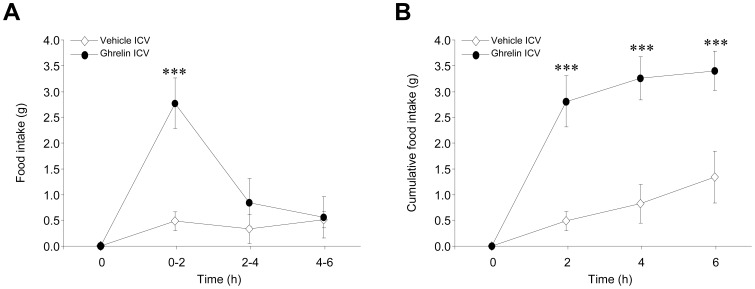
Effect of central ghrelin administration on food intake. (**A**) Two-hour interval and (**B**) cumulative food intake of rats ICV treated with vehicle or ghrelin for 6 hours. ***P<0.001 *vs.* vehicle ICV. All data are expressed as mean ± SEM.

**Figure 2:**
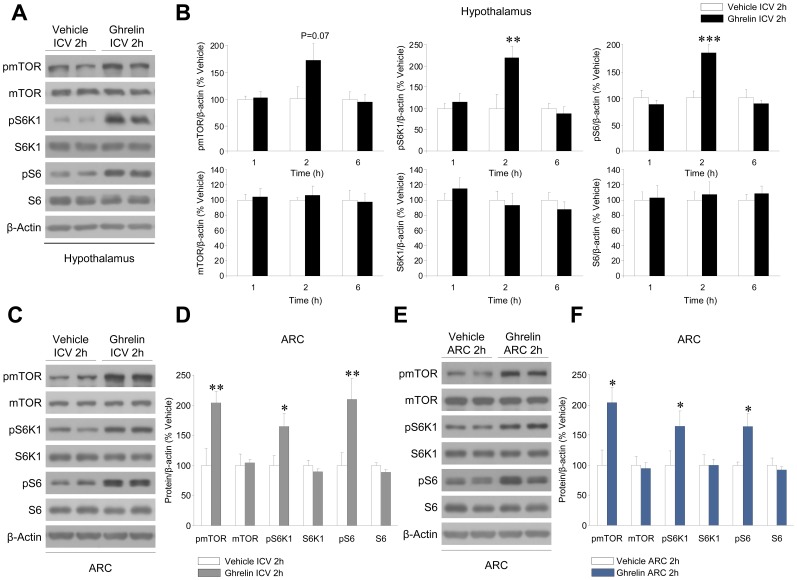
Effect of central ghrelin administration on hypothalamic mTOR pathway. (**A, C and E**) Western blot autoradiographic images and (**B**) hypothalamic or (**D and F**) ARC protein levels of the different proteins of the mTOR pathway in vehicle or ghrelin treated rats. For the ICV injections and analysis of the total hypothalamic protein extracts (**A and B**) rats were treated during 1, 2 and 6 hours. For the ICV and stereotaxic injections in the ARC followed of analysis of the ARC protein extracts (**A and B**) rats were treated during 2 hours. *P<0.05, **P<0.01, ***P<0.001 *vs.* vehicle (ICV or ARC). All data are expressed as mean ± SEM.

ICV administration of ghrelin exerted a marked orexigenic action, which was evident 2 hours after injection and was consistent after 6 hours **(** **Figures 1A and 1B**). Recent evidence has linked hypothalamic mTOR signaling to modulation of feeding [20]–[28], thus we aimed to investigate the effect of central ghrelin on this metabolic pathway. Our data showed that ghrelin induced a marked activation of mTOR signaling, as demonstrated by increased protein levels of phosphorylated (active) mTOR (pmTOR) at Ser2448, and its downstream targets, namely pS6K1 at Thr389 and pS6 at Ser235/236 **(** **Figures 2A and 2B** **)**. In all cases, that stimulatory effect was transient (2 hours without significant effect at 1 and 6 hours) and associated with no changes in the total amount of mTOR, S6K1 and S6 protein levels **(** **Figures 2A and 2B** **)**.

### Central Administration of Ghrelin Activated mTOR Pathway in the ARC

Next, we aimed to identify the hypothalamic nucleus where ghrelin exerted its action on the mTOR pathway. Firstly, we analyzed the mTOR pathway in the ARC of rats ICV treated with ghrelin. Our data recapitulated the same effect detected when analyzing the total hypothalamic protein levels, with ICV ghrelin inducing a marked activation of mTOR signaling in the ARC **(** **Figures 2C and 2D** **).** Secondly, to add further anatomical insight to our studies we analyzed mTOR signaling in the ARC of rats stereotaxically treated with ghrelin in that nucleus. Our data showed again that mTOR pathway was activated in the ARC following ghrelin injection **(** **Figures 2E and 2F** **)**. These data demonstrate that ghrelin exerts a marked stimulatory effect on hypothalamic mTOR pathway and that this effect is, at least partially, located in the ARC.

### Inhibition of Hypothalamic mTOR Reversed the Orexigenic Effect of Ghrelin

**Figure 3:**
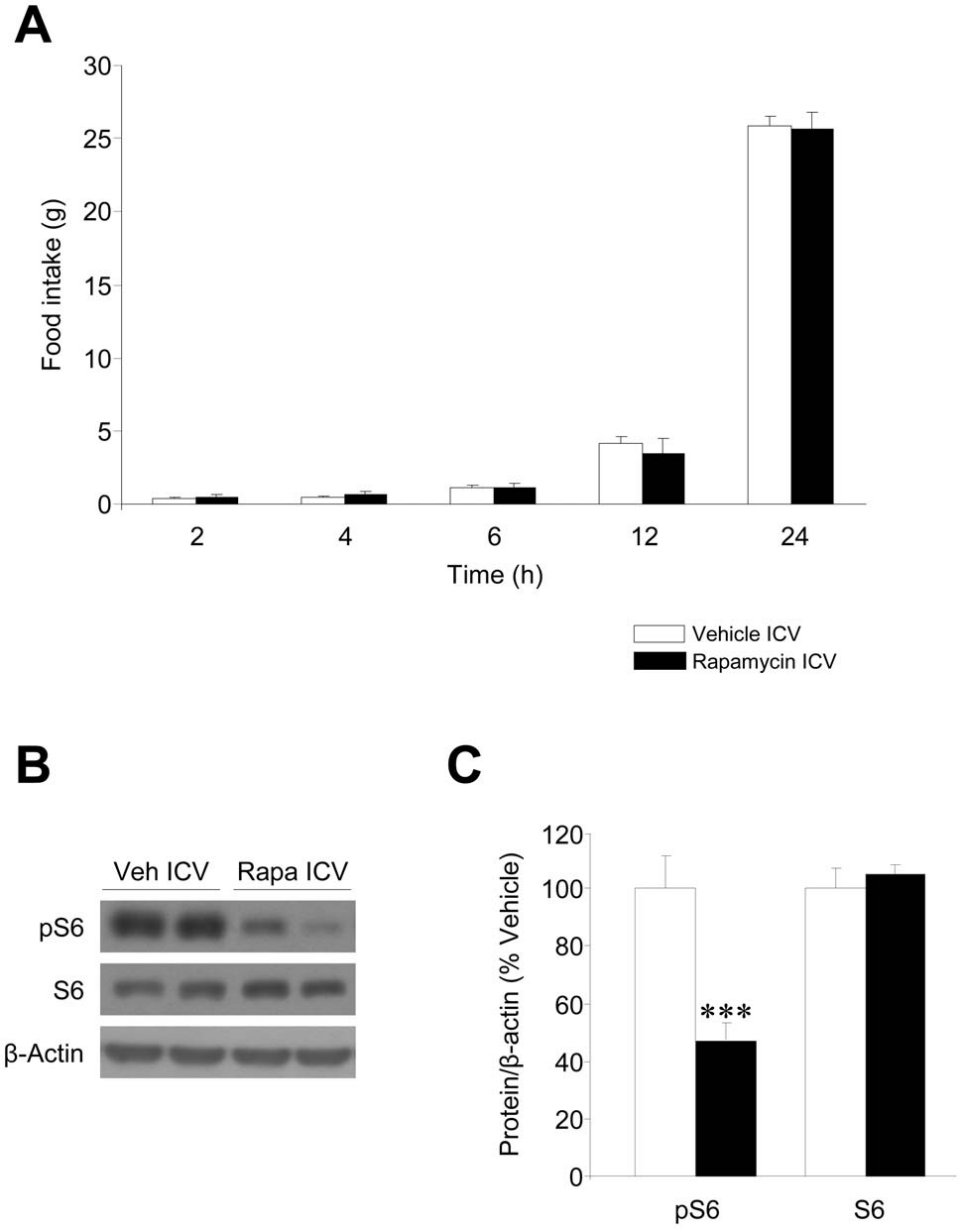
Effect of central administration of rapamycin on food intake and hypothalamic mTOR signaling. (**A**) Food intake, (**B**) Western blot autoradiographic images and hypothalamic protein levels of S6 and pS6 of rats ICV treated vehicle or rapamycin. ***P<0.001 *vs.* vehicle ICV. All data are expressed as mean ± SEM.

**Figure 4:**
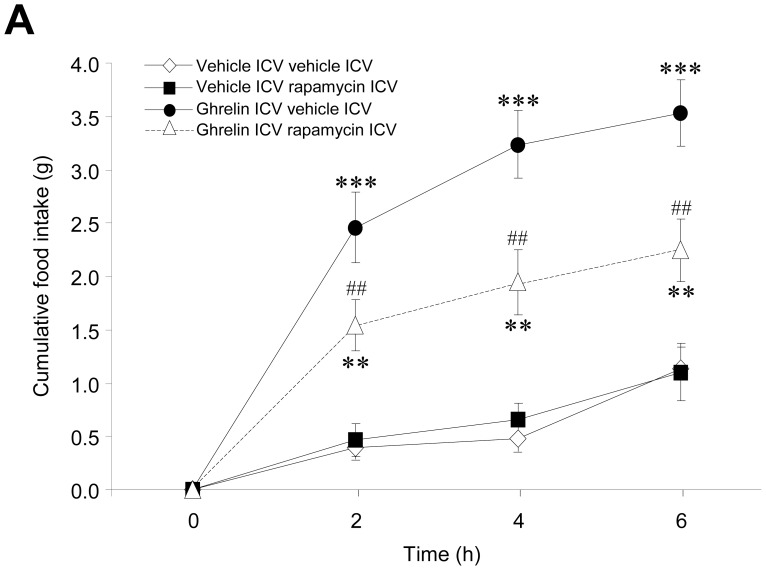
Effect of central administration of rapamycin on the orexigenic effect of ghrelin. (**A**) Food intake of vehicle or ghrelin ICV treated rats, previously ICV treated with vehicle or rapamycin. **P<0.01, ***P<0.001 *vs.* vehicle ICV vehicle ICV; ##*P*<0.01 *vs.* ghrelin ICV vehicle ICV. All data are expressed as mean ± SEM.

To determine the existence of any mechanistic link between ghrelin’s orexigenic effect and the activation of mTOR signaling, we investigated the effects of rapamycin, an inhibitor of mTOR [28], on ghrelin action. The selected dose of rapamycin [20], [28] induced neither an anorectic effect *per se* at any evaluated time (**Figure 3A**), nor taste aversion, illness or malaise (data not shown) [28], but inhibited hypothalamic mTOR signaling, as demonstrated by significantly reduced levels of pS6 (**Figures 3B and 3C**). Although this dose of rapamycin was sub-effective when injected alone, our data showed that ICV injection of rapamycin decreased the orexigenic effect of ghrelin at all the evaluated time points (2, 4 and 6 hours) (**Figure 4A**).

### Inhibition of Hypothalamic mTOR Signaling Prevented the Ghrelin-induced Increase in AgRP and NPY, as well as their Upstream Transcription Factors

**Figure 5:**
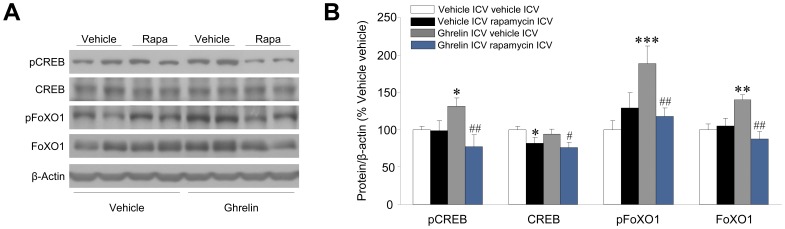
Effect of central rapamycin administration on ghrelin’s action on hypothalamic transcription factors. (**A**) Western blot autoradiographic images and (**B**) hypothalamic protein levels of hypothalamic transcription factors in vehicle or ghrelin ICV treated rats, previously ICV treated with vehicle or rapamycin. *P<0.05, **P<0.01, ***P<0.001 *vs.* vehicle ICV vehicle ICV; #*P*<0.05, ##*P*<0.01 *vs.* ghrelin ICV vehicle ICV. All data are expressed as mean ± SEM.

**Figure 6:**
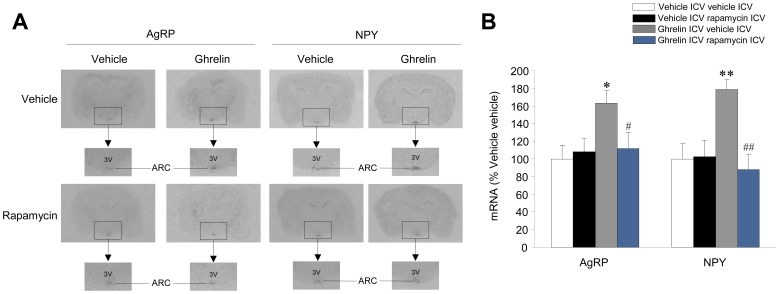
Effect of central rapamycin administration on ghrelin’s action on AgRP and NPY mRNA expression. (**A**) *In situ* hybridization autoradiographic images and (**B**) AgRP and NPY mRNA levels in the arcuate nucleus of the hypothalamus (ARC) of vehicle or ghrelin ICV treated rats, previously ICV treated with vehicle or rapamycin. *P<0.05, **P<0.01 *vs.* vehicle ICV vehicle ICV; #*P*<0.05, ##*P*<0.01 *vs.* ghrelin ICV vehicle ICV; 3V: third ventricle. All data are expressed as mean ± SEM.

Having shown that the central inhibition of mTOR signaling decreased ghrelin’s orexigenic action, we investigated the effect of this treatment on the expression levels of AgRP, NPY and their transcription factors. Our results showed that administration of rapamycin prevented the ghrelin-induced increase in pCREB, FoXO1 and pFoXO1 (**Figures 5A and 5B**), as well as AgRP and NPY (**Figures 6A and 6B**) in rats. These data demonstrate that ghrelin’s effects on *Agrp* and *Npy* gene expression, as well as their upstream transcription factors are mediated, at least partially, by a mTOR signaling-dependent mechanism.

## Discussion

In this study we first demonstrate that the orexigenic effect of ghrelin requires an intact hypothalamic mTOR pathway and that this action elicits increased levels of AgRP and NPY mRNA expression in the ARC, through modulation of the transcription factors pCREB, FoXO1 and pFoXO1.

Current evidence has demonstrated that ghrelin’s orexigenic effect is mediated by the selective modulation of hypothalamic SIRT1/p53/AMPK and fatty acid metabolism pathways, as well as ROS levels, which ultimately increase AgRP and NPY expression in the ARC [2], [4]–[10]. Although it is clear that the aforementioned molecular mechanism is a *bona fide* component of ghrelin signaling, it is totally unclear whether alternative metabolic pathways could mediate ghrelin-induced increases to food intake. At the hypothalamic level, recent evidence supports the concept that mTOR signaling plays a pivotal role in the networks regulating energy homeostasis [20]–[28]. Additionally, it was reported that ghrelin and mTOR signaling are interacting at peripheral level during changes in nutritional status [38]. Furthermore, inhibition of the gastric mTOR signaling by rapamycin stimulated the expression of gastric ghrelin mRNA in mice, whereas the activation of the gastric mTOR signaling by L-leucine decreased the expression of gastric ghrelin [38]. All these evidence have led us to investigate the possible involvement of the hypothalamic mTOR pathway on ghrelin's action.

Our data show that central ghrelin administration promotes a marked increase in the phosphorylated (active) form of mTOR and its downstream targets, pS6K1 and p6 in the ARC. Therefore, we hypothesized that increases in AgRP and NPY levels after ghrelin administration, and subsequent hyperphagia, might be mediated by specific modulation of hypothalamic mTOR signaling, a notion also supported by data showing that mTOR is expressed in AgRP/NPY neurons in the ARC [20], [39]. Our results show that inhibition of mTOR signaling following rapamycin treatment negates the orexigenic action of ghrelin treatment. Of note, this action is associated with normalization of AgRP and NPY expression in the ARC, as well as the two key transcription factors modulating their expression, pCREB and FoXO1 (both total and phosphorylated forms). Overall, these results indicate that ghrelin-induced food intake (at 2 hours and further time) is mediated by hypothalamic mTOR signaling and that this effect is placed in the ARC. These results are in agreement with a recent report showing activation of S6 in the ARC after central treatment with ghrelin or fasting [40], a situation where circulating levels of total ghrelin are increased [41].

Although a large quantity of data during the last decade have shown that hypothalamic mTOR signaling plays a major role in the modulation of energy homeostasis [20]–[28], questions remain regarding the final output of hypothalamic mTOR activation. Initially, it was proposed that activation of mTOR signaling in the hypothalamus promoted anorexia; in fact it was demonstrated that short-term central administration of anorectic factors, such as leucine, leptin, CNTF, α-lipoic acid or BMP7 increase hypothalamic mTOR signaling [20], [22], [23], [26], [29], [40]. However, long-term activation of hypothalamic mTOR signaling elicits an orexigenic response, indicated by the following evidence. Mice lacking tuberous sclerosis protein 1 (TSC1, also called hamartin, a major upstream negative regulator of mTOR) in the hypothalamus (TSC1 KO), as well as mice with specific deletion of TSC1 within POMC neurons (POMC-TSC1 KO), develop hyperphagia due to activation of mTOR [25]. Importantly, as evidenced in our ghrelin paradigm, both phenotypes are totally reversed by rapamycin administration. In keeping with these data, we have recently reported that thyroid hormone-induced feeding, both short-term (central administration) and long-term (hyperthyroidism), is mediated by selective modulation of the mTOR pathway in the ARC, which elicits increased expression of AgRP and NPY [28]. Succinctly, our data demonstrate that both thyroid hormone and ghrelin promote feeding via activation of mTOR signaling in the ARC.

The reasons for these divergent effects may not be related to the timing (short-term *vs.* long-term activation), since in our experiments ghrelin was administrated over a short time period (2–6 hours). Alternatively, in our view, as recently proposed [28], [40], the differences may be related to the existence of nucleus-specific or even neuron-specific responses. In this sense, it has been reported that leptin activates mTOR signaling in AgRP neurons in the ARC but, in contrast, decreases mTOR activity in the VMH [40]. Analogously, in a similar way to ghrelin, thyroid hormone increases mTOR signaling in the ARC (but not in the VMH) [28]. It therefore seems feasible that the respective activation of the mTOR pathway by ghrelin and anorectic factors, such as leptin, may involve different intracellular events or even interactions with alternative intracellular transduction pathways to mediate orexigenic or anorexigenic responses, a hypothesis that will require further investigation. In any event our data further highlight the relevance of cellular sensors, notably AMPK and mTOR, in the neuroendocrine control of energy homeostasis. In this regard, the data reported here, as well as previous work assessing the role of AMPK [5], [7], [42] may indicate that the orexigenic effect of ghrelin is mediated by acting in two different hypothalamic nuclei. At the VMH the effect of ghrelin is mediated by AMPK with the subsequent alterations in lipid metabolism [5], [7], [42] while at the ARC is mediated by mTOR. Further work with selective silencing of the respective two cellular sensors in these neuronal populations is needed in order to fully prove this hypothesis. Whatever the case, it is clear that ghrelin's orexigenic action is a very well preserved physiological mechanism, as it requires a complex and redundant molecular pathway in the hypothalamus, to ultimately increase AgRP and NPY expression (transcription, translation and secretion), and subsequently promote feeding.

In summary, our study shows that mTOR activation is a hypothalamic, mainly ARC-located, mechanism mediating ghrelin’s action on feeding through increased *Agrp* and *Npy* gene expression. Our data also describe activation of hypothalamic mTOR signaling as a mediator of food intake, of potential importance for the understanding and treatment of obesity.
